# Impact of Docosahexaenoic Acid on Gene Expression during Osteoclastogenesis *in Vitro*—A Comprehensive Analysis

**DOI:** 10.3390/nu5083151

**Published:** 2013-08-13

**Authors:** Masako Akiyama, Ken-ichi Nakahama, Ikuo Morita

**Affiliations:** Department of Cellular Physiological Chemistry, Graduate School, Tokyo Medical and Dental University, 1-5-45, Yushima, Bunkyo-ku, Tokyo 113-8549, Japan; E-Mails: m-akiyama.cell@tmd.ac.jp (M.A.); morita.cell@tmd.ac.jp (I.M.)

**Keywords:** polyunsaturated fatty acid, docosahexaenoic acid, osteoclast

## Abstract

Polyunsaturated fatty acids (PUFAs), especially *n*-3 polyunsaturated fatty acids, docosahexaenoic acid (DHA) and eicosapentaenoic acid (EPA), are known to protect against inflammation-induced bone loss in chronic inflammatory diseases, such as rheumatoid arthritis, periodontitis and osteoporosis. We previously reported that DHA, not EPA, inhibited osteoclastogenesis induced by the receptor activator of nuclear factor-κB ligand (sRANKL) *in vitro*. In this study, we performed gene expression analysis using microarrays to identify genes affected by the DHA treatment during osteoclastogenesis. DHA strongly inhibited osteoclastogenesis at the late stage. Among the genes upregulated by the sRANKL treatment, 4779 genes were downregulated by DHA and upregulated by the EPA treatment. Gene ontology analysis identified sets of genes related to cell motility, cell adhesion, cell-cell signaling and cell morphogenesis. Quantitative PCR analysis confirmed that DC-STAMP, an essential gene for the cell fusion process in osteoclastogenesis, and other osteoclast-related genes, such as Siglec-15, Tspan7 and Mst1r, were inhibited by DHA.

## 1. Introduction

Clinical studies previously demonstrated that *n*-3 polyunsaturated fatty acid (PUFA), especially docosahexaenoic acid (DHA), concentration in serum or plasma was positively associated with bone or periodontal disease, such as peak bone mineral density in the total body [1] and femoral neck bone mineral density in women [2], and may be affected by the severity of periodontal disease [3]. Moreover, PUFAs have been considered as drug candidates for various diseases, because they do not cause severe side effects. The beneficial effects of PUFAs on infant allergies, cancer therapy, atherosclerosis and Alzheimer’s disease have been reported [4,5,6,7]. However, PUFAs did not exhibit significant effects in some clinical trials [8,9]. Thus far, fish oil or DHA/eicosapentaenoic acid (EPA) capsules, a mixture of DHA and EPA, have been used for the ingestion of *n*-3 PUFA. To clarify the effect and mechanism of each PUFA, comparative studies need to be conducted using purified PUFAs. Although the effects of DHA and EPA have been considered to be similar, recent findings have suggested differences between them. For example, DHA, but not EPA, reduced ambulatory blood pressure and heart rate in humans [10,11]. We showed that DHA strongly inhibited sRANKL-induced osteoclastogenesis *in vitro*, whereas EPA enhanced it [12]. The mechanisms by which PUFAs affect osteoclastogenesis have also been examined. Zhu *et al*. reported that resolvin E1 (a metabolite of EPA, RvE1) inhibited osteoclast fusion by downregulating DC-STAMP in bone marrow macrophages (BMMs) [13]. On the other hand, our previous results suggested that PGE_3_, a metabolite of EPA, accelerated osteoclast fusion. Therefore, the effects of EPA on osteoclastogenesis may be explained by the effect of another metabolite of EPA, such as RvE1 or PGE_3_.

In this study, we examined the gene expression profiles of BMMs, which were cultured with or without sRANKL in the presence or absence of DHA. We then compared genes regulated either by DHA or EPA. We also identified genes related to osteoclastogenesis that were affected by DHA.

## 2. Materials and Methods

### 2.1. Mouse Bone Marrow Macrophage (BMM) Culture and the Induction of Osteoclast Formation

Specific pathogen-free male ddY mice (6–8 weeks) were purchased from Japan SLC (Shizuoka, Japan). Mice bone marrow cells were obtained by flushing the femurs and tibias using a 17-gauge needle with alpha modified eagle minimum essential Medium (α-MEM, GIBCO/Invitrogen, Carlsbad, CA, USA) supplemented with 10% fetal calf serum (FCS; Biowest, Nuaillé, France). After the red blood cells were lysed using 0.83% ammonium chloride solution, the mononuclear cells obtained were passed through a 40 μm cell strainer (BD Falcon, Franklin Lakes, CA, USA) to remove debris. Cells were seeded in a 96-well plate at a density of 5 × 10^5^ cells/well with α-MEM containing 10% FCS, MEM non-essential amino acid solution (Sigma-Aldrich, St. Louis, MO, USA) and gentamicin (Nacalai Tesque, Kyoto, Japan). Osteoclast formation was induced by 10 ng/mL macrophage colony-stimulating factor (M-CSF; Peprotech, Rocky Hill, NJ, USA) and 100 ng/mL sRANKL (Oriental Yeast, Tokyo, Japan) contained in a culture medium with DHA (Cayman, Ann Arbor, MI, USA) or EPA (Cayman). DHA or EPA were dissolved in ethanol as stock solutions. Control cells were treated with ethanol as a vehicle. We previously reported that DHA and EPA significantly affected osteoclastogenesis in a dose-dependent manner [12], Ten micrometers of DHA or EPA affect osteoclast formation without cytotoxicity.

Animal handling and experimental procedures were approved by the Animal Care and Use Committee of the Tokyo Medical and Dental University.

### 2.2. Tartrate-Resistant Acid Phosphatase (TRAP) Staining

Osteoclast formation was evaluated using tartrate-resistant acid phosphatase (TRAP) staining with the formation of multiple nuclei. After the cultures were washed with phosphate buffered saline (PBS), cells were fixed with ethanol/acetone (4:1) for one minute and were then dried and stained with TRAP staining solution (50 mM sodium acetate buffer/pH 5.0, naphthol AS-BI phosphoric acid sodium salt (Sigma-Aldrich), fast red ITR salt (Sigma-Aldrich) and 10 mM sodium tartrate (Wako, Osaka, Japan)). TRAP-positive cells with three or more nuclei that were formed in the culture were considered to be osteoclasts. The area occupied by osteoclasts was determined with the low-power field (2.18 mm × 1.8 mm) of a microscope and calculated by an image processing program (ImageJ; National Institutes of Health, Bethesda, MD, USA).

### 2.3. Gene Expression Analysis Using Agilent Whole Mouse Genome Oligo Microarrays

Total RNA was extracted from BMMs 72 h after the treatment of cells with M-CSF and sRANKL in the presence of PUFAs using TRIZOL reagent (Invitrogen/Life Technologies, Carlsbad, CA, USA) or the RNeasy Plus kit (Qiagen, Venlo, The Netherlands), according to the manufacturer’s protocol (Invitrogen). Messenger RNA extraction from total RNA and measurement of the microarray assay was outsourced to Miltenyi Biotec (Bergisch Gladbach, Germany) or Takara Bio (Osaka, Japan).

### 2.4. Quantification of mRNA by Real-Time PCR

Total RNA from two or three days after the PUFA treatment of BMMs was extracted by TRIZOL reagent, according to the manufacturer’s protocol. Three micrograms of total RNA were subjected to reverse-transcription (ReverTra Ace, TOYOBO, Osaka, Japan). cDNA samples were amplified with the KAPA SYBR^®^ Fast qPCR Kit (KAPA BIOSYSTEMS, Woburn, MA, USA), and PCR cycling was performed using the ABI PRISM^®^ 7500 real-time instrument (Applied Biosystems, Carlsbad, CA, USA). PCR amplifications were performed with specific primers (Table 1). GAPDH served as an endogenous control.

### 2.5. Data Analyses

Microarray data analyses were performed with GeneSpring GX (Agilent Technology, Santa Clara, CA, USA). Normalization was performed with the percentile shift. Values were represented as the mean ± standard error of the mean (SE) for osteoclast formation and real-time PCR data. The means of different groups were used in multiple comparison testing using Dunnett’s test or Tukey-Kramer’s test correction for multiple comparisons. A *p*-value of less than 0.05 was considered significant.

**Table 1:** Primer sequences used for real-time PCR.

Gene	Primer sequence (5′–3′) positions in mRNA	Product size (bp)
GAPDH	F:TACAGCAACAGGGTGGTGGAC	233
	R:GTGGGTGCAGCGAACTTTATT	
DC-STAMP	F:AAAACCCTTGGGCTGTTCTT	399
	R:CTTCGCATGCAGGTATTCAA	
Tspan7	F:TGTAATCCTGTTACAGGTTGTGTTG	147
	R:CCACACTCACTTTTAAATTGATCTGATG	
Siglec-15	F:TACTTCTGCCGCGTGGAGTT	114
	R:CAGCACCGAGATGTTGACGA	
Mst1r	F:CACGACCCACCTTCAGAGCCCTAGT	300
	R:TTGTCCTAGGCCCAGAGGCAGCTTG	

**Figure 1:**
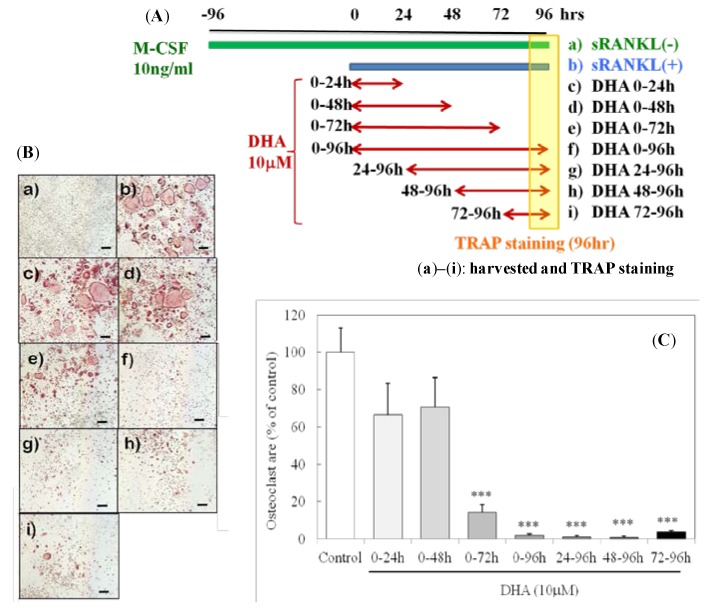
DHA inhibited sRANKL-induced osteoclast differentiation at the late stage. (**A**) Culture conditions and (**B**) representative images of osteoclasts (TRAP staining (**a**)–(**i**)). The scale bar indicates 200 μm. (**C**) The areas occupied by osteoclasts (TRAP^+^ cells with three or more nuclei) were analyzed. Each column and bar represents the mean ± SE of four to five wells. *** Significantly different from the control (sRANKL(+)); *p* < 0.0001 by Dunnett’s multiple comparison test.

## 3. Results

### 3.1. Stage-Dependent Effect of DHA on sRANKL-Induced Osteoclastogenesis

We determined whether the inhibitory effect of DHA depended on the stage of osteoclastogenesis. BMMs were treated with sRANKL and M-CSF with or without 10 μm of DHA. DHA did not inhibit osteoclastogenesis when it was applied from 0 to 48 h after the addition of sRANKL; however, a strong inhibitory effect was observed when it was applied at the late stage of osteoclastogenesis (Figure 1). These results suggested that DHA inhibited osteoclastogenesis at the late stage.

### 3.2. Effect of DHA and EPA on Osteoclast Formation

**Figure 2:**
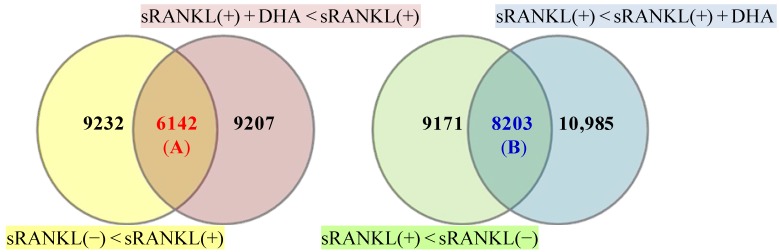
Gene expression profiles of BMMs. Venn diagrams show at least two-fold changes in genes with or without sRANKL in the presence or absence of DHA. Numbers in the Venn diagram indicate the number of genes. The yellow circle includes genes that were upregulated by sRANKL(+) compared to sRANKL(−), the red circle includes genes that were downregulated by DHA + sRANKL(+) compared with sRANKL(+), the green circle includes genes that were downregulated by sRANKL(+) compared with sRANKL(−) and the blue circle includes genes that were upregulated by DHA + sRANKL(+) compared with sRANKL(+). (**A**) Number of genes upregulated by sRANKL and inhibited by DHA; (**B**) Number of genes down-regulated by sRANKL and enhanced by DHA.

TRAP staining after the treatment of cells with M-CSF only, M-CSF and sRANKL, M-CSF and sRANKL with 10 μm DHA and M-CSF and sRANKL with 10 μm EPA. DHA strongly inhibited osteoclastogenesis; however, EPA enhanced it (Figure 2). DHA-treated BMMs were shown to be TRAP-positive, but not multinucleated. This result suggested that DHA inhibits the cell-cell fusion process during osteoclastogenesis.

### 3.3. Gene Expression Profiles of BMMs Cultured with or without sRANKL in the Presence or Absence of DHA

**Figure 3:**
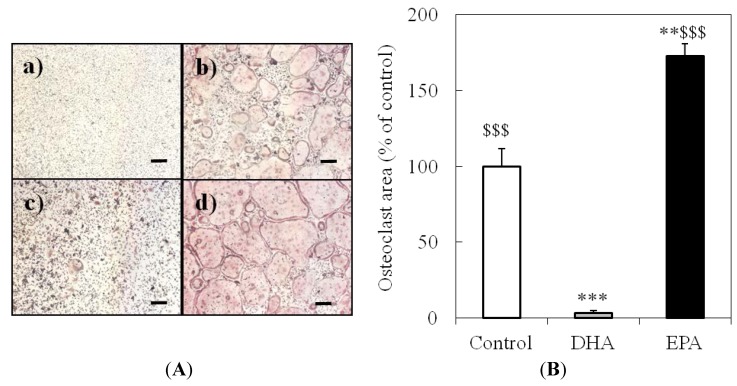
Effect of docosahexaenoic acid (DHA) and eicosapentaenoic acid (EPA) on sRANKL-induced osteoclastogenesis in bone marrow macrophages (BMMs). (**A**) Representative image of osteoclasts. BMMs were cultured without (**a**) or with (**b**–**d**) sRANKL in the presence of 10 μm DHA (**c**) or 10 μm EPA (**d**). Cells were stained for tartrate-resistant acid phosphatase (TRAP) after a 96 h culture. The scale bar indicates 200 μm. (**B**) The areas occupied by osteoclasts (TRAP^+^ cells with three or more nuclei) were analyzed. Each column and bar represents the mean ± SE of four or five wells. * Significantly different from the control (sRANKL(+)) (** *p* < 0.01, *** *p* < 0.001) by Tukey-Kramer’s multiple comparison test. $$$ Significantly different from the DHA-treated group (*p* < 0.0001) by Tukey-Kramer’s multiple comparison test.

Total RNA was extracted from BMMs 72 h after the treatment of cells with M-CSF and sRANKL with or without DHA. Among the 15,374 genes upregulated by the sRANKL treatment, 6142 genes (A) were downregulated by DHA. In contrast, among the 17,374 genes downregulated by the sRANKL treatment, 8203 genes (B) were upregulated by DHA (Figure 3). Twenty-two osteoclast differentiation-related genes were identified in 6142 genes (A), including Dcstamp, Nfatc1 and Siglec-15. On the other hand, only two genes were found in 8203 genes (B). Table 2 shows the genes that were upregulated by sRANKL, inhibited by DHA and stimulated by EPA in the second microarray experiment.

**Table 2:** Gene expression related to osteoclastogenesis.

Ontology Term	Gene	Fold change downregulated by DHA
GO:0030316	Calcr	0.43
	Dcstamp	0.44
osteoclast differentiation	Nfatc1	0.47
	Ostm1	0.45
GO:0072674	Gpr55	0.36
multinuclear osteoclast differentiation		
GO:0036035	Dcstamp	0.44
osteoclast development		
GO:0045672	Car2	0.49
positive regulation of osteoclast differentiation	Itgb3	0.50
GO:2001204	Siglec-15	0.35
regulation of osteoclast development		
GO:0045670	Esrra	0.48
regulation of osteoclast differentiation		

**Figure 4:**
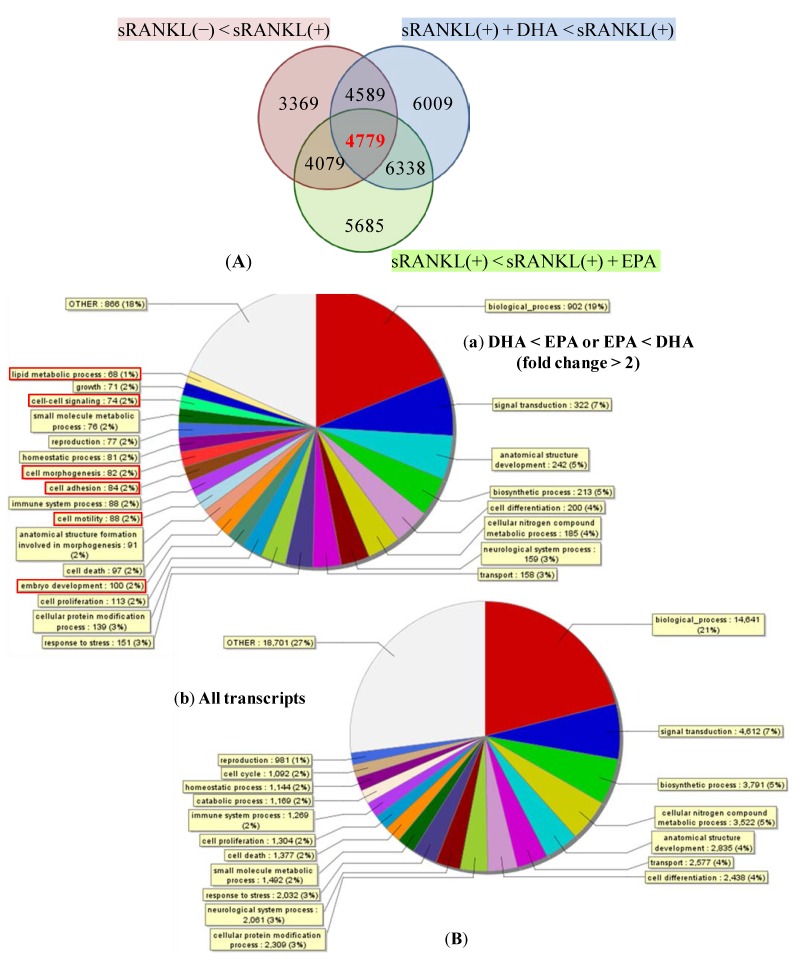
Genes significantly affected by the DHA or EPA treatment. (**A**) The Venn diagram shows the grouping of genes with at least two-fold changes. The numbers in the diagram indicate the number of genes. The red circle include genes that were upregulated by sRANKL(+) compared with sRANKL(−), the blue circle include genes that were downregulated by DHA + sRANKL(+) compared with sRANKL(+) and the green circle include genes that were upregulated by sRANKL(+) + EPA compared with sRANKL(+). (**B**) The pie chart shows GO slim annotation of the differentially expressed transcripts between DHA and EPA (**a**). All transcripts were shown in (**b**).

### 3.4. Gene ontology (GO) Enrichment on Biological Process Ontology

Total RNA was extracted from BMMs 72 h after the treatment of cells with M-CSF only, M-CSF and sRANKL, M-CSF and sRANKL with 10 μm DHA and M-CSF and sRANKL with 10 μm EPA. Overlapping depicted transcripts (4779 transcripts) were selected as the genes that were upregulated by sRANKL, inhibited by DHA and stimulated by EPA (Figure 4A). Gene ontology was analyzed with Generic GO slim developed by the GO Consortium. These genes were classified into GO slim terms and showed the term including more than 10 genes (Figure 4B). The following GO slim terms were enriched in the gene group that exerted differences in expression between DHA and EPA: “embryo development”, “cell motility”, “cell adhesion”, “cell morphogenesis”, “cell-cell signaling” and “the lipid metabolic process”.

### 3.5. Effect of DHA and EPA on Osteoclast Differentiation-Related Genes

**Figure 5:**
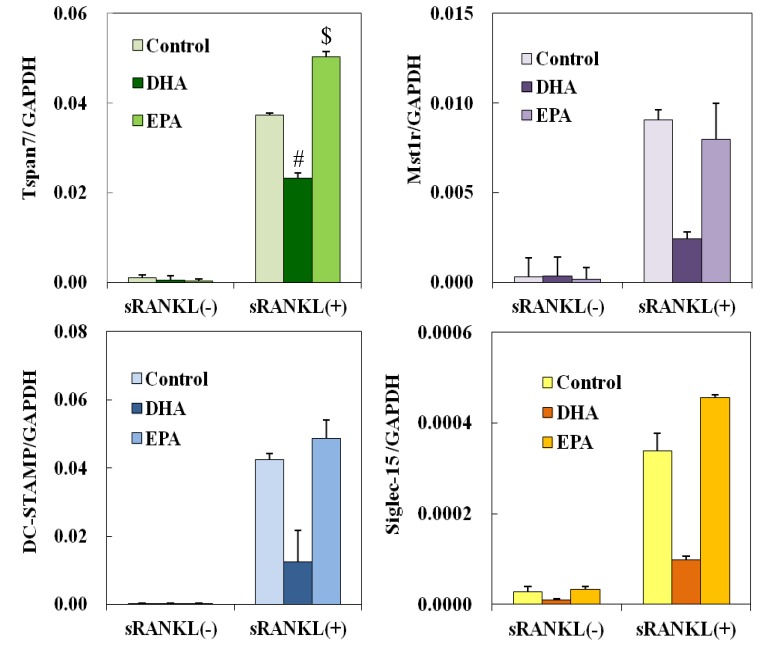
Effects of EPA on gene expression that was attenuated by the DHA treatment during osteoclastogenesis. DC-STAMP, Tspan7, Siglec-15 and Mst1r mRNA levels were normalized to that of GAPDH. Each column and bar represents the mean ± SE of three mice. # Significantly different from EPA-treated group (*p* < 0.05) by Tukey-Kramer’s multiple comparison test. $ Significantly different from DHA (*p* < 0.05) by Tukey-Kramer’s multiple comparison test.

Messenger RNA expression levels of the genes involved in osteoclastogenesis were examined by real-time PCR. DHA, not EPA, inhibited the expression of DC-STAMP, Tspan7, Siglec-15 and Mst1r 72 h after the treatment (Figure 5).

## 4. Discussion

DHA, a kind of *n*-3 PUFA, strongly inhibited osteoclastogenesis. We performed gene expression analysis aimed at identifying genes affected by the DHA treatment during osteoclastogenesis and showed that DHA and EPA affected gene-related embryo development, cell motility, cell adhesion, cell morphogenesis, cell-cell signaling and the lipid metabolic process.

Recent studies have shown that supplementation with PUFAs inhibited osteoporosis [2] and chronic rheumatoid arthritis [14]. According to the report that examined the mechanisms by which PUFAs inhibit osteoclastogenesis, the metabolites of PUFAs, such as prostaglandins, hydroxyeicosatetraenoic acids and leukotrienes, at least in part, directly affected osteoclastogenesis [15,16,17,18,19]. Osteoclastogenesis plays a pivotal role in bone homeostasis; bone is maintained by active remodeling through the balance between bone resorption by osteoclasts and bone synthesis by osteoblasts. Osteoclasts are bone resorbing cells and are essential for bone remodeling. Osteoclasts are formed from hematopoietic progenitors in the monocyte/macrophage lineage.

Osteoclast differentiation undergoes several steps, including progenitor growth, differentiation to mononuclear pre-osteoclasts, cell-cell fusion to multinuclear osteoclasts and the activation of bone resorption. In this study, the effect of DHA on osteoclastogenesis was stage-specific. At the late stage, BMMs treated with DHA differentiated into mononuclear TRAP-positive osteoclasts. However, DHA applied zero to 48 h after sRANKL stimulation did not inhibit osteoclastogenesis. The effect of DHA on osteoclastogenesis may be associated with the cell-cell fusion process approximately 72 h after the sRANKL treatment and may not be related to pre-osteoclast differentiation.

According to the gene expression profiling of BMMs, among 15,374 genes stimulated by sRANKL, 6142 genes were downregulated by DHA. Osteoclast-related genes, such as Dcstamp, Itgb3 and Siglec-15, belonged to group A. On the other hand, among the 17,374 downregulated genes in cells treated with sRANKL, 8203 genes were upregulated by DHA (group B). Gene ontology analysis between EPA- and DHA-treated groups identified 25 differentially expressed groups, including embryo development, cell motility, cell adhesion, cell morphogenesis, cell-cell signaling and the lipid metabolic process.

Dendritic cell-specific transmembrane protein (DC-STAMP) is reported to be an essential molecule for the fusion process of pre-osteoclasts [20]. In this study, a decrease in DC-STAMP by the DHA treatment may have been responsible for the inhibitory effect of DHA on osteoclastogenesis. Promoter analysis showed that NFATc1, AP-1 PU.1, Tal1 and MITF were involved in the regulation of Dcstamp [21,22]. Another gene involved in group A was NFATc1, a master regulator of osteoclastogenesis. The mechanism by which DHA inhibits NFATc1 expression is still unclear in this study. Interestingly, Siglec-15 was shown to play an important role in RANKL-RANK-NFATc1 signaling to form a complex with DNAX-activating protein 12 kDa (DAP 12) and Syk [23]. The downregulation of Siglec-15 may cause the attenuation of NFATc1 autoamplification by reducing Syk-Ca2^+^ signaling [24]. Tspan7, also called transmembrane 4 superfamily member 2 (TMSF2), is a member of the tetraspanin family of proteins. Iwai *et al.* reported that some of the Tspan superfamily proteins were expressed in osteoclast precursors and osteoclasts and that Tspan5 contributed to cell-cell fusion during osteoclastogenesis [25]. Tspan7 was recently shown to form a complex with proteins interacting with C-kinase-1 (PICK1) [26]. Moreover, PKCα and calcineurin were identified as interacting proteins with PICK1, as predicted by a flexible docking approach [27]. PKCα and CaMKIIα have been identified as PICK1 binding proteins [28]. The disruption of these protein complexes may contribute to the inhibitory effect of DHA, because PKCα and CaMKIIα were shown to play important roles in osteoclastogenesis [29,30]. No reports have shown the involvement of Mst1r, macrophage stimulating 1 receptor, in osteoclastogenesis; however, osteoclast activity was stimulated by receptor activation (Kurihara *et al.* [31]). The inhibitory effect of DHA on the expression of DC-STAMP, Siglec-15, Tspan7 and Mst1r was confirmed by real-time PCR. The expression of Tspan7 and Siglec-15 was inhibited by DHA, but was stimulated by EPA. The expression of DC-STAMP and Mst1r was inhibited by DHA, but was unaffected by EPA. Further investigations into the interaction of those genes will reveal the mechanism for the inhibitory effect of DHA on osteoclastogenesis.

## 5. Conclusions

This study showed that DHA inhibited osteoclastogenesis, which was related to cell-cell fusion and not osteoclast precursors. Gene expression profiling of BMMs in sRANKL-induced osteoclastogenesis showed that DHA and EPA affected gene-related embryo development, cell motility, cell adhesion, cell morphogenesis, cell-cell signaling and the lipid metabolic process. DC-STAMP, Siglec-15, Tspan7 and Mst1r expression was downregulated by DHA, but not EPA. These findings may contribute to the molecular understanding of the beneficial effects of DHA as a food supplement.
